# Contraceptive use following unintended pregnancy among Ugandan women living with HIV

**DOI:** 10.1371/journal.pone.0206325

**Published:** 2018-10-25

**Authors:** Jana Jarolimova, Jerome Kabakyenga, Kara Bennett, Winnie Muyindike, Annet Kembabazi, Jeffrey N. Martin, Peter W. Hunt, Yap Boum, Jessica E. Haberer, David R. Bangsberg, Angela Kaida, Lynn T. Matthews

**Affiliations:** 1 Department of Medicine, Massachusetts General Hospital, Boston, MA, United States; 2 Mbarara University of Science and Technology, Mbarara, Uganda; 3 Bennett Statistical Consulting, Ballston Lake, NY, United States; 4 University of California at San Francisco, San Francisco, CA, United States; 5 Epicentre, Médicins sans Frontières (MSF), Yaounde, Cameroon; 6 Center for Global Health and Department of Medicine, Massachusetts General Hospital; Harvard Medical School, Boston, MA, United States; 7 Oregon Health and Science University-Portland State University School of Public Health, Portland, OR, United States; 8 Simon Fraser University, Faculty of Health Sciences, Burnaby, British Columbia, Canada; 9 Center for Global Health and Division of Infectious Disease, Massachusetts General Hospital, Boston, MA, United States; South Eastern Kenya University, KENYA

## Abstract

**Background:**

Preventing unintended pregnancy is critical for women living with HIV (WLWH) to safely achieve their reproductive goals. Family planning services should support WLWH at risk of repeat unintended pregnancies. We examined the relationship between unintended pregnancy and subsequent contraception use among WLWH in Uganda.

**Study design:**

This was a retrospective analysis of data from a longitudinal cohort of individuals initiating antiretroviral therapy (ART), restricted to women with pregnancy (confirmed via urine β-hcg testing) between 2011–2013. The exposure of interest was intended vs unintended pregnancy, and the outcome was self-report of modern contraceptive use (hormonal methods, intrauterine device, sterilization, and/or consistent condom use) at 12 (range 6–18) months post-partum. A log-binomial model was used to estimate relative risks of modern contraceptive use post-partum based on intent of the index pregnancy, adjusted for age, socioeconomic status, education, relationship and HIV status of pregnancy partner, contraceptive use prior to pregnancy, years since HIV diagnosis, ART regimen, and CD4 cell count.

**Results:**

Among 455 women, 110 women reported 110 incident pregnancies with report on intent. Women had a baseline median age of 29 years, baseline CD4 count 403 cells/mm^3^, and were living with HIV for 3.8 years. Fifty pregnancies (45%) were reported as unintended and 60 (55%) as intended. Postpartum, 64% of women with unintended and 51% with intended pregnancy reported modern contraception (p = 0.24). In adjusted models, there was no association between pregnancy intent and post-partum contraception. However, contraceptive use prior to the referent pregnancy was positively associated with post-partum contraceptive use (aRR 1.97 (95% CI 1.12–3.48, p = 0.02), while higher baseline CD4 cell count was associated with lower post-partum contraceptive use (aRR 0.95, 95% CI 0.90–0.99, p = 0.02).

**Conclusions:**

Almost half of incident pregnancies among WLWH in this cohort were unintended. Experiencing an unintended pregnancy was not associated with post-partum contraceptive use. Creative strategies to support contraceptive uptake for birth spacing and prevention of unintended pregnancies in the post-partum period are needed.

## Background

Individuals’ freedom to decide on the number and timing of their children is central to the attainment of sexual and reproductive health and rights worldwide [1], an integral component of the international right to health [2]. The ability to achieve one’s fertility desires extends to women living with HIV (WLWH) [3], a particularly vulnerable population experiencing stigma, gender inequality[3], and high rates of maternal and post-partum mortality in sub-Saharan Africa [4–8]. Enabling WLWH to plan and space their pregnancies additionally reduces HIV transmission to children, and forms the second prong of the WHO/UNAIDS Global Plan for eliminating perinatal HIV transmission [9]. Addressing unmet need for family planning among women living with HIV (WLWH) is thus a global priority to reduce maternal mortality and perinatal transmission of HIV, and to attain the right to health for all women.

Despite these well-publicized goals, millions of women worldwide continue to have unmet family planning needs, with rates in some parts of the world, such as sub-Saharan Africa, stagnant from 1970–2010 [10]. This unmet family planning need contributes significantly to the incidence of unintended pregnancies [11], with the balance primarily a result of contraceptive failure[12]. In turn, unintended pregnancies, historically defined by intent reported by women prior to conception [13], have significant health consequences for mothers and their children [14,15]. Unmet family planning need, while not a reflection of lack of contraceptive access alone[16], is an important measure for the targeting of contraceptive services.

Women in Uganda, with one of the highest fertility rates in the world at 5.4 births per woman [17], and a national HIV prevalence of 7% [18], have significant unmet family planning needs. Over 30% of unmarried, sexually-active Ugandan women report unmet family planning needs[17], and 44% of pregnancies in Uganda are reported as unintended [19]. Studies from elsewhere in sub-Saharan Africa report 34–67% of all pregnancies as unintended, often with higher rates among WLWH than among women living without HIV [20–24]. Modern contraceptive use among Ugandan WLWH has remained low; reported rates primarily range between 40% and 45%, though isolated studies have reported higher rates of up to 74% [25–28] [29], with unintended pregnancy being common [25]. Better integration and access to comprehensive family planning services is thus needed to achieve HIV prevention and treatment goals [30] and to promote sexual and reproductive rights of WLWH.

Unmet need refers both to need for limiting (for women who do not desire more children) and spacing (for women who ideally wait 24 months prior to conceiving again) pregnancies[10]. In both cases, the post-partum period is an important time to identify and address unmet family planning need. Data from higher-income country settings suggest that unintended pregnancy is associated with an increased risk of subsequent unintended pregnancy[31]. Most post-partum WLWH in Uganda do not desire pregnancy in the near future [32], however, rates of unmet family planning need in the post-partum period among Ugandan women reported in 2001 were over 70% [33]. Identifying women at increased risk for repeat unintended pregnancy may be an important opportunity to effectively provide focused family planning services in large HIV clinics. Recent data from a retrospective study in Kenya suggest that women with a preceding unintended pregnancy are more likely to be using contraception in the post-partum period[34], however, it is unknown whether the same is true for WLWH. WLWH with unintended pregnancies may similarly recognize their fertility after an unintended pregnancy and thus better assess their risk of subsequent unintended pregnancies; conversely, structural barriers that impeded their access to contraceptive use in the first place may continue to limit their use of modern contraception in the post-partum period. We postulated that intent of an index pregnancy may identify WLWH at higher risk of repeat unintended pregnancy and thus in need of contraceptive services in the post-partum period. Using data from a cohort study of WLWH in rural Uganda, we examined whether women reporting an unintended pregnancy were more or less likely to report use of modern contraception at 12 months post-partum.

## Methods

### Study sample

Study participants were enrolled in the Uganda AIDS Rural Treatment Outcomes (UARTO) cohort study, which ran from 2005 to 2015. Clients initiating ART at an HIV clinic in Southwestern Uganda, at least 18 years old, and living within 60 kilometers of the clinic were eligible to enroll. Contraceptive services were not directly available at the HIV clinic site, but were available, free of charge, through the family planning clinic at the same hospital campus. Participants completed baseline and approximately quarterly interviews and phlebotomy. Interviewer-administered questionnaires detailed socio-demographics, sexual behavior, and partner dynamics, including partner HIV status. Laboratory data included CD4 cell count measurements and HIV-RNA, and clinical records indicated ART regimen.

This analysis utilized data from the Reproductive Health Component of this cohort, initiated in October 2011 with follow-up through April 2014. This annual questionnaire assessed sexual and reproductive health and history, fertility desire, contraceptive use, partner HIV status, and partner fertility desire; the questionnaire was also administered with incident pregnancy (up to quarterly). Among women of reproductive age, pregnancy was assessed quarterly with urine β-Hcg testing.

All women of reproductive age (18–49 years old) with a positive pregnancy test between 2011–2013 (i.e, during the reproductive health component of UARTO) and who completed at least one post-partum reproductive health survey were eligible for inclusion in this analysis. Women who were not tested with urine β-Hcg but self-reported pregnancy were considered pregnant. Women who reported pregnancy but had a negative urine β-Hcg were not considered pregnant.

### Measures

The exposure of interest was the intent of the index pregnancy, determined using questions derived from the Pregnancy Risk Assessment Monitoring System (PRAMS) developed and validated by the U.S. Centers for Disease Control (CDC) [35], measured at time of detection of incident pregnancy on quarterly urine β-Hcg testing (called the “pregnancy visit” for this analysis). Unintended pregnancy was defined as either mistimed or unwanted pregnancy[15]. A pregnancy was considered unintended if the participant reported that she “wanted to be pregnant later”, or did “not want to be pregnant then or at any time in the future”, or replied “no” when asked if she was “trying to get pregnant”; all other non-missing responses (not including “Don’t know/don’t remember” and “refused”) were considered intended.

The primary outcome was self-reported use of modern contraception at 12 months post-partum (called “the post-partum visit” for this analysis). Since most women did not have a visit at exactly 12 months post-partum, we considered visits within 6–18 months after birth outcome, based on extrapolation to a 9-month pregnancy (dates of pregnancy outcome were missing for most participants). Sensitivity analyses expanded this window to range from 6 to 36 months from referent pregnancy.

Modern contraceptive use was derived at this post-partum visit from self-report of a modern contraceptive method[36] (oral contraceptive pills, injections, intra-uterine device, sub-dermal implant, female sterilization, primary male partner sterilization, and/or consistent condom use with all partners) within the 6 months preceding the visit. Participants with repeat pregnancy at the post-partum visit were classified according to self-reported contraceptive use, to account for either discontinuation of use prior to conception or contraceptive failure. Since contraceptive failure would only be expected to account for a minority of repeat pregnancies[12] and the contraception question asked newly-pregnant women about their contraceptive use in the past 6 months, we conducted a sensitivity analysis where pregnant participants were categorized as not using contraception.

Covariates obtained at the time of the initial Reproductive Health Survey included age, socioeconomic status (Filmer Pritchett Asset Index[37]), education level, number of prior live births, relationship with pregnancy partner, HIV status of pregnancy partner, modern contraceptive use in the 6 months prior to index pregnancy, and years since HIV diagnosis. CD4 cell count and HIV-RNA samples were collected and measured. Efavirenz-containing ART regimen was also considered, since women would have been counseled to avoid pregnancy while on efavirenz due to concerns about teratogenicity existing at the time of data collection. The first Reproductive Health Survey did not coincide with the enrollment visit for participants enrolled prior to October 2011. Data on contraceptive use prior to index pregnancy was obtained from the Reproductive Health Survey completed at the time of pregnancy detection. Participants were asked about contraceptive use in the preceding 6 months or since the last reproductive health questionnaire was completed, whichever was shorter. Covariates obtained from the post-partum visit included outcome of index pregnancy, post-partum fertility desire, primary partner fertility desire at the pregnancy visit and at the postpartum visit.

### Statistical methods

A log-binomial model was used to obtain relative risks of modern contraceptive use post-partum based on intention of the index pregnancy. This model was separately adjusted for the covariates of interest. Covariates with a significant effect on outcome at a level of p<0.05 were then incorporated into serial adjusted models. Given the relatively small sample size and inconsistent missingness across variables, several smaller models were evaluated rather than one large model.

### Ethical considerations

This study was approved by the ethics review boards of Partners Healthcare, the Mbarara University of Science and Technology, and Simon Fraser University. Administrative approvals were secured from the President’s Office and the Ugandan National Council for Science and Technology.

## Results

Among 455 women of reproductive age who completed the Reproductive Health Component of the study, there were 120 incident pregnancies during 1,161 woman-years of follow-up. Pregnancy intent was reported for 110 of these pregnancies; 50 (45%) reported as unintended, and 60 (55%) as intended. Of the 110 pregnancies, 26 resulted in a live birth, 5 in miscarriage, 0 in stillbirth, 2 had other pregnancy outcomes, and 78 were missing data on pregnancy outcome. Table 1 summarizes baseline characteristics of these 110 participants at time of first Reproductive Health Survey and at time of the first detection of index pregnancy. Overall, compared with women with intended pregnancies, women with unintended pregnancies were older (median age 29.9 vs 28.8 years), had a higher pre-ART CD4 cell count (458 vs 380 cells/mm^3^), and were more likely to report that their primary partner either “did not want the current pregnancy then or at any time” (26% vs 3%) or “didn’t care or don’t know” (26% vs 5%).

**Table 1:** Characteristics of women living with HIV receiving ART who became pregnant over the study follow-up.

Variable, N (for women with variable complete at referent study visit)	Women with unintended pregnancy, n = 50	Women with intended pregnancy, n = 60
**At first reproductive health survey**		
**Age, years, n = 110**	29.9 (24.7,34.3)	28.8 (25.1,34.3)
**Number of living children, n = 94**		
0	5 (12.5%)	11 (20%)
1	9 (22.5%)	9 (17%)
2	6 (15%)	16 (30%)
3 or 4	9 (22.5%)	12 (22%)
5 or more	11 (27.5%)	6 (11%)
**HIV viral load, n = 39**		
<400 copies/uL	15 (94%)	22 (96%)
**Asset Index**^§^ **Quintile, n = 94**		
1	10 (25%)	8 (5%)
2	9 (23%)	7 (13%)
3	10 (25%)	14 (26%)
4	6 (15%)	11 (21%)
5	5 (13%)	14 (26%)
**Years since HIV diagnosis, n = 109**	3.8 (1.0, 5.8)	3.8 (0.7,6.7)
**CD4 (cells/mm**^**3**^**), n = 110**	458 (343,629)	380 (282,464)
**At first detection of pregnancy**		
**Relationship with pregnancy partner, n = 110**		
Spouse/legal partner	35 (70%)	40 (67%)
Regular partner	14 (28%)	17 (28%)
One-time encounter/Ongoing casual	1 (2%)	3 (5%)
**Modern contraceptive use prior to pregnancy, n = 59**		
No	13 (48%)	14 (44%)
Yes	14 (52%)	18 (56%)
**Reported primary partner fertility desire, n = 110**		
Sooner or Then	20 (40%)	52 (87%)
Later	4 (8%)	3 (5%)
Not want pregnancy then or any time	13 (26%)	2 (3%)
Didn't care or Don't know	13 (26%)	3 (5%)

^§^Filmer-Pritchet Asset Index^22^

Data presented as n (%) or median (IQR) unless otherwise noted.

At the post-partum visit, 85% of women with unintended and 67% of women with intended referent pregnancy reported not wanting any more children in the future (Table 2). 62% of women with unintended vs 38% of women with intended pregnancy reported that their primary male partner definitely or probably does not want them to have another child.

**Table 2:** Pregnancy outcome, and participant and primary partner fertility desire as reported at 6–18 months post-partum (n = 82).

	Women with unintended pregnancy	Women with intended pregnancy
**Personal future fertility desire at the post-partum visit, n = 72**		
Like to have another child	5(15%)	9(23%)
Not like to have another child	28(85%)	26(67%)
Undecided/Don't know	0	4(10%)
**Partner fertility desire at postpartum visit (as reported by participant), n = 77**		
Definitely or probably yes	12(32%)	19(48%)
Definitely or probably not	23(62%)	15(38%)
Never discussed or don't know	2(5%)	6(15%)
**Outcome of index pregnancy, n = 33**		
Live Birth	15(79%)	11(79%)
Miscarried	3(16%)	2(14%)
Other pregnancy outcome*	1(5%)	1(7%)

*‘Other pregnancy outcome’ indicates any outcome other than live birth, miscarriage, or stillbirth.

**Fig 1:**
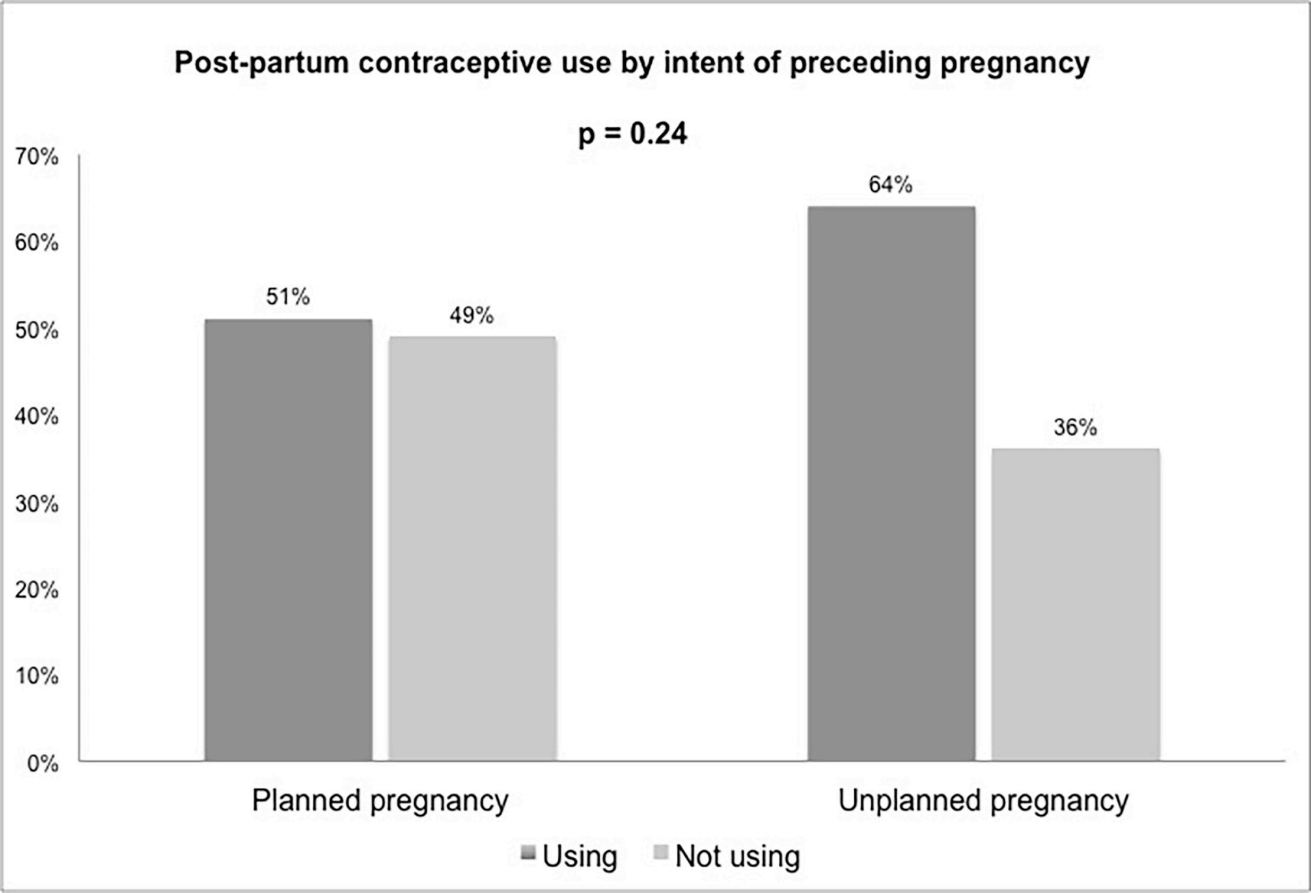
Proportion of women reporting modern contraceptive use at 6–18 months post-partum, by intent of referent pregnancy (n = 82). 51% of women with a referent intended pregnancy and 64% of women with a referent unintended pregnancy were using a modern contraceptive method at the post-partum visit. The overall rate of modern contraceptive use at the post-partum visit was 57%.

**Fig 2:**
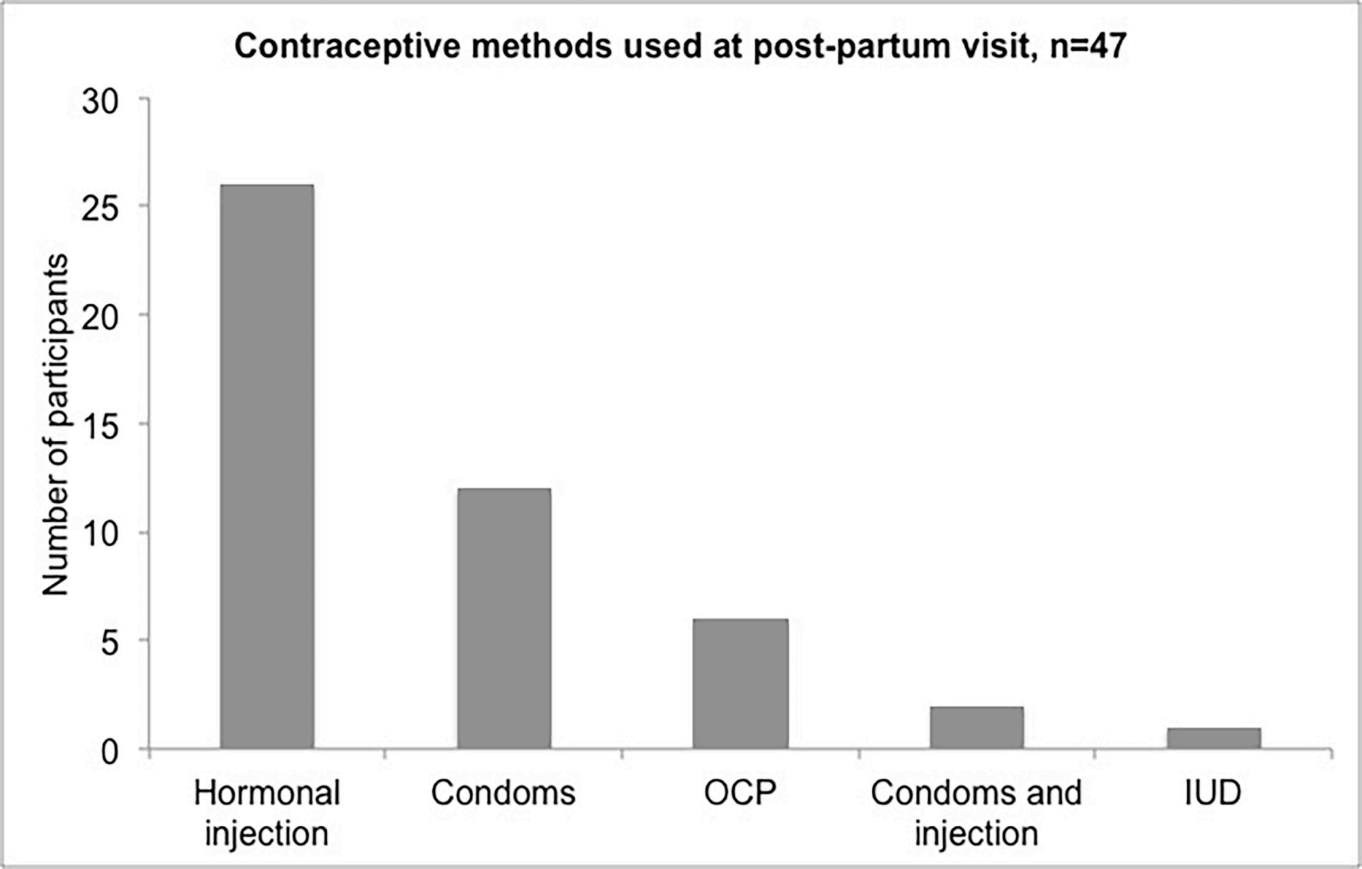
Contraceptive methods reported at the post-partum visit.

Data on the primary outcome, contraceptive use 6–18 months post-partum, was available for 82 participants. The post-partum visit occurred a median of 11.2 months (range 6–18) post-partum. Of these, 47 (57%) reported modern contraceptive use postpartum, the proportion was 64% among women with an unintended and 51% with a referent intended pregnancy (Fig 1). Of the women reporting contraceptive use post-partum, 26 (55%) were using injections, 12 (26%) condoms alone, 6 (13%) OCPs alone, 2 (4%) condoms and injections, and 1 (2%) IUD **(**Fig 2**).** At the time of the post-partum visit, 10 women had a repeat positive pregnancy test, among whom 8 reported contraceptive use (5 reporting injections, 3 reporting OCPs).

Unadjusted analysis revealed a lack of relationship between referent pregnancy intent and modern contraceptive use post-partum (RR 0.80 (95% CI 0.55,1.16), p = 0.24, Table 3). In adjusted models, contraceptive use prior to index pregnancy was associated with increased post-partum contraceptive use with aRR 1.97 (95% CI 1.12–4.38, p = 0.02), while higher baseline CD4 count was associated with lower contraceptive use, with aRR 0.95 (95%CI 0.90–0.99, p = 0.02) for every 50 cells/mm^3^ increase (Table 3), when adjusted for referent pregnancy intent.

**Table 3:** Relative risk of reported modern contraception use at 6–18 months post-partum (PP)*^¶^.

**Model**	**Covariates**	**RR (95% CI)**	**p-value**
Unadjusted (n = 82)	Intended vs. unintended	0.80 (0.55,1.16)	0.24
Adjusted models	Covariates	aRR (95% CI)	p-value
1. Intended plus prior modern contraception (n = 41)	Intended vs unintended	1.34 (0.87,2.05)	0.18
	**Prior Contraception vs. not using modern contraception prior to pregnancy**	**1.97 (1.12,3.48)**	**0.02**
2. Intended plus baseline CD4 cell count (n = 82)	Intended vs unintended	0.74 (0.52,1.04)	0.09
	CD4 count (per 50 cells/mm^3^)	0.95 (0.90–0.99	0.02
3. Intended plus post-partum desire future children (n = 68)	Intended vs unintended	0.77 (0.50,1.18)	0.23
	Participant wants more vs. Does not want more children	1.32 (0.84,2.07)	0.23
4. Intended plus partner future fertility desire (n = 77)	Intended vs unintended	0.73 (0.48,1.11)	0.14
	Partner “definitely/probabaly DOES” want more children vs “Never discussed/DNK”	0.65 (0.361.18)	0.16
	Definitely/probably DOES NOT” vs. “Never discussed/DNK”	0.68 (0.38,1.24)	0.21
DNK = do not know			

*Covariates without a significant association with post-partum contraceptive use include age, education, socioeconomic status, relationship with pregnancy partner, HIV status of pregnancy partner, primary partner pregnancy desire, years since HIV diagnosis, efavirenz-containing regimen, birth outcome, postpartum fertility desire, and primary partner post-partum fertility desire.

¶ The unadjusted association between intent of index pregnancy and post-partum contraceptive use was subsequently adjusted for covariates of interest; covariates with a significant effect on outcome at p<0.05 were incorporated into serial adjusted models.

Models did not show an association of post-partum contraceptive use with participants’ age, socioeconomic status, education level, years since HIV diagnosis, efavirenz-containing ART regimen, or participant’s relationship with or HIV status of the pregnancy partner. Self-reported personal or partner fertility desire at the time of the post-partum visit was similarly not associated with post-partum contraceptive use.

Sensitivity analysis classifying 10 women with repeat pregnancies at the post-partum visit as not using effective contraception did not alter the results of the primary analysis: intent of referent pregnancy was not associated with post-partum contraceptive use (RR 0.86 95% CI 0.55–1.36, p = 0.52). Similarly, expanding the post-partum visit window to include all visits within 6–36 months after the initial pregnancy visit did not meaningfully alter the results.

## Discussion

In this cohort of WLWH in rural Uganda, 45% of pregnancies were unintended. Intent of referent pregnancy was not associated with effective post-partum contraception use. Overall, 57% of participants reported modern contraception at 12 months post-partum, despite 75% of women not wanting to have children at that time. Among those using contraception, over half were using injectable hormonal methods.

The reported rate of unintended pregnancy in our cohort (45%) is similar to that reported nationally for Uganda in 2011 (44%)[19]. Studies in sub-Saharan Africa have found similar rates of unintended pregnancy among WLWH [21–23], though several report rates over 60% [20,24]. Given increased maternal mortality for WLWH, family planning counseling and service provision for these women remains crucial.

In this study, 64% of women with referent unintended and 51% with intended pregnancy reported modern contraceptive use at 12 months post-partum, with an overall post-partum contraceptive use rate of 57%. These results are somewhat higher than overall contraceptive use reported among WLWH at this site [28,38], and higher than rates among sexually-active unmarried (44%) and married (26%) women in Uganda as a whole in 2011 [19]. Increased contact with the healthcare system through routine HIV care, antenatal, and peri-partum care may have contributed to higher rates of contraceptive use in the post-partum period this cohort. At the time of this study, contraceptive services were not directly provided in conjunction with HIV care at the study site, however, subsequent efforts have been made to integrate HIV and family planning services.

Referent pregnancy intent was not associated with contraception use. It is possible that there is a true lack of association between pregnancy intent and post-partum contraceptive use in this cohort. A similar absence of correlation was noted in a study of adolescent girls living with HIV in Kenya[39], in which a significant proportion of participants had experienced repeat pregnancies before age 19. It could also be measurement error: without a survey tool specific to sub-Saharan Africa, the pregnancy intent questions may not accurately identify unintended pregnancy. Women may in fact want more children due to social expectations, but feel pressured to report that they either did not want their preceding pregnancies, do not want future children, or are using contraception, due to a history of stigmatization of childbearing for WLWH[40]. In addition, the small sample size may limit power to detect differences.

Post-partum contraceptive use was not associated with participant fertility desire in the post-partum period, in contrast to a previous study from this same site examining contraceptive use[41]. Three quarters of women reported at the post-partum visit that they would not like to have another child; slightly higher than rates reported elsewhere in sub-Saharan Africa [20,21]. Whether this is due to ongoing stigmatization of pregnancy for WLWH in this setting or the relatively-older age of this cohort is unclear. Nevertheless, the discrepancy between fertility desire and the contraceptive use rate of 57% demonstrates persistent unmet need for family planning in this cohort.

These data highlight the importance of efforts to focus on strengthening contraceptive counseling and utilization in the postpartum period. This was a quantitative study that did not explore women’s self-reported reasons for not using contraception, therefore, comprehensive recommendations regarding increased utilization of post-partum contraception in this population is beyond the scope of this study. However, the findings shed light on several areas in which contraceptive care provision for this population could be improved. Integration of HIV care delivery and contraceptive services is an evidence-based approach to increasing access to contraception for women living with and without HIV in sub-Saharan Africa [21,42]. Integration of family planning services with HIV care is currently being implemented at our study site, with the effect of improved access and service provision on pregnancy intentions and contraceptive use among post-partum WLWH being studied (https://clinicaltrials.gov/ct2/show/study/NCT02964169).

Contraceptive use prior to index pregnancy and baseline CD4 count were the only significant determinants of post-partum contraceptive use. The association with prior contraceptive use suggests that women who were more likely to be using contraception prior to conception, due to either better access or better education, were again more likely to be using contraception post-partum, irrespective of whether their intervening pregnancy was intended or not. While the numbers in this study are small, this finding suggests that WLWH with incident pregnancies should be assessed for contraceptive use in the period prior to pregnancy. Those reporting no contraceptive use in the pre-conception period should be focused on for additional counseling regarding post-partum contraceptive uptake. The reasons for the statistically significant (albeit small) association between baseline CD4 cell count and post-partum contraceptive use are not clear. One potential reason may be that women entering the cohort with lower CD4 cell counts were more frequently engaged in care due to advanced disease, thus they were exposed to a greater amount of counseling and health education, and may have therefore been more likely to use contraception in the post-partum period.

In sum, data from this cohort of WLWH in Uganda suggests that eliciting intent of an index pregnancy using currently-available tools does not predict effective post-partum contraceptive use. A stronger emphasis in counseling on post-partum contraceptive use and prevention of repeat unintended pregnancies should be incorporated into care for WLWH. WLWH who are pregnant should be assessed for contraceptive use in the pre-conception period, and those who report not using contraceptive methods prior to their current pregnancy should be supported to access post-partum contraception commensurate with their reproductive goals. Access to long-acting and injectable methods of contraception should be ensured in this setting. The development of a validated tool to assess pregnancy intent among women living with HIV in sub-Saharan Africa should be further explored.

There are several limitations to this study. Determination of pregnancy intent is challenging without a current, culturally-appropriate, validated questionnaire, and women may be misreporting pregnancy intent or future fertility desire due to social desirability bias in the healthcare setting. There is potential for reporting bias leading to the underestimation of rates of unintended pregnancy, since women may be less likely to report a pregnancy as unintended once they are pregnant. Data on outcomes and covariates were not available for all study participants, leading to decreased power to detect significant differences in contraceptive use by pregnancy intent. Some covariates (e.g. pregnancy outcome) were not included in models due to missing data. Data on post-partum contraceptive use were not available for all incident pregnancies, however, rates of missing data were similar for women with referent unintended and intended pregnancies.

## Summary

Understanding contraceptive use and fertility desire after unintended pregnancies can provide an opportunity for focused interventions to address unmet family planning needs among women of reproductive age with HIV in Uganda. This exploratory study, with the objective of better understanding the relationship between unintended pregnancy and subsequent contraceptive use, aims to inform targeted contraceptive counseling for post-partum women living with HIV in sub-Saharan Africa.

## Abbreviations

HIV: Human immunodeficiency virus
WLWH: women living with HIV
ART: anti-retroviral therapy
OCP: oral contraceptive pill
IUD: intrauterine device
LARC: long-acting reversible contraception
